# Pharmacodynamics and Biodistribution of Single-Dose Liposomal Amphotericin B at Different Stages of Experimental Visceral Leishmaniasis

**DOI:** 10.1128/AAC.00497-17

**Published:** 2017-08-24

**Authors:** Andrew A. Voak, Andy Harris, Zeeshan Qaiser, Simon L. Croft, Karin Seifert

**Affiliations:** a Faculty of Infectious and Tropical Diseases, Department of Immunology and Infection, London School of Hygiene & Tropical Medicine, London, United Kingdom; b Pharmidex, London, United Kingdom

**Keywords:** biodistribution, drug potency, pharmacodynamics, visceral leishmaniasis

## Abstract

Visceral leishmaniasis is a neglected tropical disease that causes significant morbidity and mortality worldwide. Characterization of the pharmacokinetics and pharmacodynamics of antileishmanial drugs in preclinical models is important for drug development and use. Here we investigated the pharmacodynamics and drug distribution of liposomal amphotericin B (AmBisome) in Leishmania donovani-infected BALB/c mice at three different dose levels and two different time points after infection. We additionally compared drug levels in plasma, liver, and spleen in infected and uninfected BALB/c mice over time. At the highest administered dose of 10 mg/kg AmBisome, >90% parasite inhibition was observed within 2 days after drug administration, consistent with drug distribution from blood to tissue within 24 h and a fast rate of kill. Decreased drug potency was observed in the spleen when AmBisome was administered on day 35 after infection, compared to day 14 after infection. Amphotericin B concentrations and total drug amounts per organ were lower in liver and spleen when AmBisome was administered at the advanced stage of infection and compared to those in uninfected BALB/c mice. However, the magnitude of difference was lower when total drug amounts per organ were estimated. Differences were also noted in drug distribution to L. donovani-infected livers and spleens. Taken together, our data suggest that organ enlargement and other pathophysiological factors cause infection- and organ-specific drug distribution and elimination after administration of single-dose AmBisome to L. donovani-infected mice. Plasma drug levels were not reflective of changes in drug levels in tissues.

## INTRODUCTION

Visceral leishmaniasis (VL) is a vector-borne neglected tropical disease (NTD) caused by protozoan parasites of the genus Leishmania. VL has a high mortality rate if untreated. Current estimates suggest 200,000 to 400,000 cases and 20,000 to 40,000 deaths per year worldwide, with over 90% of cases occurring in India, Bangladesh, Sudan, South Sudan, Brazil, and Ethiopia (1).

Major clinical manifestations of disease are fever, enlargement of the liver and spleen, weight loss, and pancytopenia. Available treatment options include pentavalent antimonials, paromomycin in combination with pentavalent antimony (in East Africa), miltefosine and amphotericin B in different formulations (2). Liposomal amphotericin B (AmBisome) has emerged as the preferred treatment for VL in Europe and the United States (3) and South Asia (2). In India, a cure rate of 95.7% was achieved with a single infusion of 10 mg/kg of AmBisome in a trial carried out at an urban referral center (4). The feasibility of this treatment regimen was confirmed in a rural setting in Bangladesh at the primary health care level (5). Single-dose AmBisome was also one component in a recent trial of short-course multidrug treatment regimens for VL in India (6).

AmBisome was initially developed to improve efficacy and reduce toxicity of amphotericin B as an antifungal agent. Its favorable therapeutic index is attributed to prolonged circulation times due to a small liposome size, allowing distribution to tissues, and a tight association of amphotericin B with the liposome bilayer, providing stability under physiological conditions (7). AmBisome also remains the antileishmanial agent with the widest therapeutic window for treatment of VL (3).

Earlier studies have established the preclinical pharmacokinetics (PK) of AmBisome in mice, rats, rabbits, and dogs (7, 8). In addition, a comparative, single-time-point biodistribution study in Leishmania donovani-infected and uninfected BALB/c mice showed that amphotericin B concentrations in liver and spleen were lower in infected mice after intravenous (i.v.) administration of AmBisome (9). A separate study showed that AmBisome, when administered as a single dose to L. donovani-infected BALB/c mice, was less effective in the chronic stage of infection compared to the acute stage (10). In anti-infective therapy with drugs that directly kill pathogens, such as amphotericin B, drug efficacy is driven by infection site-specific drug concentrations (11, 12). We therefore hypothesized that the decrease in potency of AmBisome observed in chronic experimental VL (EVL) is linked to a decrease in organ-specific drug concentrations associated with progression of infection. Here we show that (i) decreased potency of AmBisome during progressive EVL is most pronounced in the spleen, (ii) total drug amounts in liver and spleen progressively decrease during EVL, (iii) organ-specific drug distribution and elimination patterns occur in EVL, and (iv) changes in drug levels in tissues are not reflected in the blood compartment.

## RESULTS

### Efficacy of single-dose liposomal amphotericin B in L. donovani-infected BALB/c mice at two different time points after infection.

Single-dose AmBisome was administered at doses of 10, 2.5, and 0.6 mg/kg of body weight 14 or 35 days after infection. Parasite burden was evaluated 7 days after dosing. A reproducible decrease in drug efficacy between day 21 and day 42 postinfection was noted in the spleen, which was more pronounced at the lower doses. In the liver, decreased drug efficacy at the lower two doses was noted in one experiment, whereas in another experiment, an increase in drug efficacy was observed at the lowest dose (Table 1).

**TABLE 1:** Efficacy of single-dose AmBisome in L. donovani-infected BALB/c mice^*a*^

Parameter	Dose (mg/kg)	Result for parameter shown					
		Liver			Spleen		
		Day 21 p.i.	Day 42 p.i.	Significance	Day 21 p.i.	Day 42 p.i.	Significance
% of reduction in parasite burden							
Expt 1	10	99 ± 0	98 ± 1	NS	99 ± 0	83 ± 4	*P* ≤ 0.05
	2.5	78 ± 2	78 ± 5	NS	53 ± 6	5 ± 5	*P* ≤ 0.0001
	0.6	21 ± 8	41 ± 4	*P* ≤ 0.01	44 ± 6	4 ± 4	*P* ≤ 0.0001
Expt 2	2.5	81 ± 2	80 ± 3	NS	71 ± 2	36 ± 11	*P* ≤ 0.05
Expt 3	10	100 ± 0	99 ± 1	NS	99 ± 0	93 ± 2	NS
	2.5	94 ± 1	80 ± 5	*P* ≤ 0.05	40 ± 6	45 ± 7	NS
	0.6	54 ± 3	22 ± 7	*P* ≤ 0.0001	38 ± 10	16 ± 9	NS
Parasite burden (LDU) in untreated control groups							
Expt 1	NA	966 ± 66	518 ± 48	*P* ≤ 0.01	88 ± 8	118 ± 15	NS
Expt 2	NA	415 ± 32	176 ± 11	*P* ≤ 0.001	18 ± 1	53 ± 8	*P* ≤ 0.01
Expt 3	NA	682 ± 54	194 ± 44	*P* ≤ 0.001	18 ± 2	84 ± 9	*P* ≤ 0.001

a Parasite burden was evaluated 7 days after drug administration on day 21 or 42 postinfection (p.i.). Data are presented as mean ± SEM. NS, not significant; NA, not applicable; LDU, Leishman-Donovan units.

### Time-kill studies.

**FIG 1:**
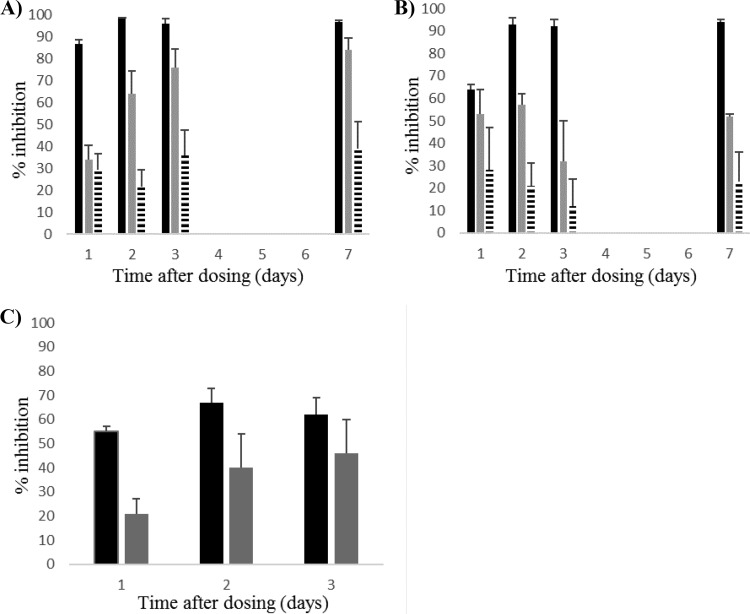
Time to kill of single-dose AmBisome in L. donovani-infected BALB/c mice. (A and B) Percentages of inhibition of parasite burden in liver (A) and spleen (B) 1, 2, 3, and 7 days after a single dose of AmBisome at dose levels of 10 mg/kg (black bars), 2.5 mg/kg (gray bars), and 0.6 mg/kg (striped bars). (C) Percentage of inhibition of parasite burden in liver (black bars) and spleen (gray bars) 1, 2, and 3 days after a single dose of 2.5 mg/kg AmBisome. Treatment was given 14 days (A and B) or 33 days (C) after infection. Data are presented as group mean (*n* = 5), and error bars represent the standard error of the mean (SEM). Data in panels A and B are representative of two separate experiments.

Single-dose AmBisome was administered to L. donovani-infected BALB/c mice 14 days after infection at doses of 10, 2.5, and 0.6 mg/kg. Parasite burden was evaluated 1, 2, 3, or 7 days after dosing. In the liver, effective parasite kill (>90% inhibition) was observed 2 days after administration of the highest dose. At the dose of 2.5 mg/kg, the hepatic parasite burden was inhibited by >60% at 2 days, >70% at 3 days, and >80% (maximum kill) at 7 days after drug administration. The dose of 0.6 mg/kg was ineffective (<40% inhibition), and no change in parasite kill was observed over time (Fig. 1A). A similar pattern of effective kill was observed in the spleen after administration of 10 mg/kg AmBisome (>80% and >90% inhibition in repeat experiments at 2 days and >90% inhibition at 3 days after drug administration), and a static pattern (<40% inhibition) was observed after administration of 0.6 mg/kg. Maximum kill at the dose of 2.5 mg/kg varied from 57 to 71% inhibition in repeat experiments, with peaks at 2 and 3 days after drug administration (Fig. 1B). In an additional experiment, L. donovani-infected BALB/c mice were treated with a single dose of 2.5 mg/kg AmBisome 33 days after infection. The hepatic parasite burden was inhibited by 55, 67, and 62%, respectively, on days 1, 2, and 3 after drug administration. In the spleen, parasite kill was low on day 1 after drug administration (<40% inhibition) and increased on days 2 and 3 after drug administration to a maximum of 46% inhibition (Fig. 1C).

### Plasma pharmacokinetics of single-dose AmBisome in L. donovani-infected BALB/c mice.

Single-dose AmBisome was administered at dose levels of 10, 2.5, and 0.6 mg/kg 14 days after infection. Plasma samples were obtained at 5 and 30 min and 1, 3, 6, and 24 h after drug administration, followed by determination of drug concentrations and estimation of PK parameters. The volume of distribution (*V*) and clearance (CL) increased with decreasing doses of AmBisome. The *V* increased from 104 ml/kg at 10 mg/kg to 252 and 681 ml/kg at 2.5 mg/kg and 0.6 mg/kg, respectively. CL was 27 ml/h/kg at a dose of 10 mg/kg and 48.1 and 41.9 ml/h/kg at doses of 2.5 and 0.6 mg/kg. The area under the concentration-time curve from 0 to 24 h (AUC_0–24_) was highest at 10 mg/kg (368.5 h · μg/ml) and lower at doses of 2.5 mg/kg and 0.6 mg/kg (50.5 and 10.8 h · μg/ml, respectively). PK parameters are summarized in Table 2.

**TABLE 2:** Pharmacokinetic profile of single-dose AmBisome in plasma of L. donovani-infected BALB/c mice^*a*^

Parameter	Result for parameter at single i.v. dose shown		
	10 mg/kg	2.5 mg/kg	0.6 mg/kg
*t*_1/2_ (h)	3.4	5.0	NQ
*T*_max_ (h)	0.08	0.08	0.08
*C*_max_ (μg/ml)	240.3	29.8	3.3
AUC_last_ (h · μg/ml)	368.5	50.5	10.8
CL (ml/h/kg)	27.0	48.1	41.9
*V* (ml/kg)	104	252	681

a Mice had been infected for 14 days at the time of dosing. The parasite burdens in untreated controls (mean ± SD) were 644 ± 25 LDU in the liver and 17 ± 1 LDU in the spleen (*n* = 3). Data are representative of two separate experiments (*n* = 1 mouse/time point). NQ, not quoted.

### Tissue distribution of amphotericin B in L. donovani-infected BALB/c mice following administration of AmBisome at two different time points after infection.

Single-dose AmBisome was administered 14 or 35 days after infection. Amphotericin B concentrations in plasma, liver, and spleen were measured 7 days after dosing. Lower amphotericin B concentrations were observed in livers and spleens when equal drug doses were administered on day 35 compared to day 14 postinfection. When expressed as nanograms per gram of tissue, drug concentrations in the liver were 2.6-, 2.2-, and 5.6-fold lower, respectively, on day 42 compared to day 21 postinfection at AmBisome doses of 10, 2.5, and 0.6 mg/kg. Respective tissue concentrations in the spleen were 4.5-, 2.8-, and 9.6-fold lower, respectively. Statistical significance was noted for the 10-mg/kg dose group (*P* ≤ 0.0001 for liver and *P* ≤ 0.01 for spleen). Organ weights significantly increased from day 21 to day 42 postinfection (*P* ≤ 0.0001 in all dose groups), with a 1.5-fold increase in the liver and 2.3- to 2.9-fold increases in the spleen. When taking these increases in organ weights into account and estimating the total drug amount per organ, the difference between amphotericin B concentrations on day 21 and day 42 postinfection decreased. Differences in total amphotericin B amounts per organ at AmBisome doses of 10, 2.5, and 0.6 mg/kg, respectively, were 1.7-, 1.5-, and 3.8-fold in the liver and 1.5-, 1.3-, and 3.9-fold in the spleen. Statistical significance was noted for the 10-mg/kg dose group in the liver (*P* ≤ 0.0001). Overall, higher amphotericin B concentrations were measured in the liver compared to the spleen. Data are summarized in Table 3. Amphotericin B concentrations in plasma remained unchanged when drug was administered on day 14 compared to day 35 postinfection. A 0.3-fold difference was noted in the 0.6-mg/kg dose group but was not statistically significant (Table 4).

**TABLE 3:** Tissue concentrations of amphotericin B in L. donovani-infected BALB/c mice 7 days after administration of single-dose AmBisome^*a*^

Parameter and AmBisome treatment group	Result for parameter at AmBisome dose shown							
	Liver		Spleen		Ratio for day 21 vs 42 p.i.		Significance for day 21 vs 42 p.i.	
	Day 21 p.i.	Day 42 p.i.	Day 21 p.i.	Day 42 p.i.	Liver	Spleen	Liver	Spleen
Amphotericin B concn (ng/g tissue) at dose:								
10 mg/kg	115,733 ± 19,582	44,232 ± 6,900	19,233 ± 8,600	4,266 ± 1,399	2.6	4.5	*P* ≤ 0.0001	*P* ≤ 0.01
2.5 mg/kg	12,220 ± 1,308	5,492 ± 862	1,770 ± 412	624 ± 205	2.2	2.8	NS	NS
0.6 mg/kg	1,430 ± 923	257 ± 58	490 ± 146	51 ± 12	5.6	9.6	NS	NS
Organ wt (mg) at dose:								
10 mg/kg	998 ± 79	1,532 ± 98	170 ± 35	492 ± 66	0.7	0.3	*P* ≤ 0.0001	*P* ≤ 0.0001
2.5 mg/kg	1,068 ± 51	1,544 ± 86	288 ± 29	654 ± 70	0.7	0.4	*P* ≤ 0.0001	*P* ≤ 0.0001
0.6 mg/kg	1,070 ± 82	1,580 ± 67	286 ± 23	698 ± 50	0.7	0.4	*P* ≤ 0.0001	*P* ≤ 0.0001
Estimated amt of amphotericin B (ng/organ) at dose:								
10 mg/kg	114,548 ± 12,631	67,456 ± 9,000	3,071 ± 699	2,046 ± 455	1.7	1.5	*P* ≤ 0.0001	NS
2.5 mg/kg	12,907 ± 1,308	8,494 ± 1,504	522 ± 79	417 ± 171	1.5	1.3	NS	NS
0.6 mg/kg	1,524 ± 985	404 ± 85	138 ± 29	35 ± 8	3.8	3.9	NS	NS

a Amphotericin B concentrations were determined 7 days after drug administration on day 21 or day 42 postinfection (p.i.) in livers and spleens (this table) and plasma (Table 4). Data are presented as group mean (*n* = 5 mice/group) ± SD. Ratios were calculated as the ratio of the mean drug concentration at day 21 p.i. to the mean drug concentration at day 42 p.i. Total amphotericin B concentrations per organ were calculated for individual mice as follows: organ weight in grams (as determined at sacrifice) × amphotericin B concentration in nanograms per gram of tissue (as measured after processing of whole organs). NS, not significant. The drug concentrations presented here were determined in the same samples used to evaluate drug potency in experiment 3 in Table 1.

**TABLE 4:** Plasma concentrations of amphotericin B in L. donovani-infected BALB/c mice 7 days after administration of single-dose AmBisome^*a*^

AmBisome treatment group	Plasma amphotericin B concn (ng/ml plasma)		Ratio for day 21 vs 42	Significance for day 21 vs 42
	Day 21	Day 42		
10 mg/kg	191 ± 23	216 ± 47	0.9	NS
2.5 mg/kg	116 ± 18	129 ± 28	0.9	NS
0.6 mg/kg	5 ± 5	16 ± 9	0.3	NS

a Amphotericin B concentrations were determined 7 days after drug administration on day 21 or day 42 postinfection (p.i.) in livers and spleens (Table 3) and plasma (this table). Data are presented as group mean (*n* = 5 mice/group) ± SD. Ratios were calculated as the ratio of the mean drug concentration at day 21 p.i. to the mean drug concentration at day 42 p.i. NS, not significant.

### Comparative amphotericin B plasma and tissue concentrations in L. donovani-infected and uninfected BALB/c mice 7 days after administration of single-dose AmBisome.

**FIG 2:**
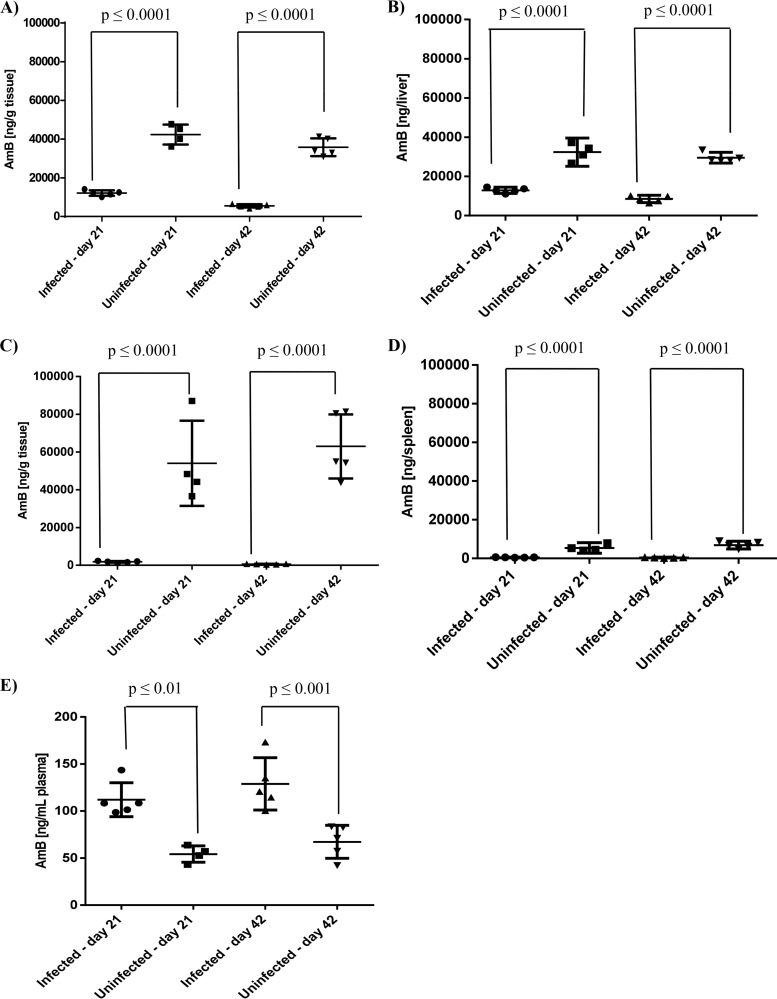
Comparative plasma and tissue drug concentrations in L. donovani-infected and uninfected BALB/c mice 7 days after administration of single-dose AmBisome. Amphotericin B (AmB) concentrations were measured on day 21 or day 42 postinfection and in uninfected BALB/c mice, maintained under identical conditions. Amphotericin B concentrations are presented as nanograms per gram of tissue (A and C) or nanograms per organ (B and D) in livers (A and B) and spleens (C and D) and as nanograms per milliliter in plasma (E). Each symbol represents data from an individual mouse. Horizontal lines indicate the mean (*n* = 4 to 5 mice/group) and error bars the standard deviation (SD). Total amphotericin B concentrations per organ were calculated for individual mice as follows: organ weight in grams (as determined at sacrifice) × amphotericin B concentration in nanograms per gram of tissue (as measured after processing of whole organs). Tissue concentrations in infected mice were measured in samples from experiment 3 in Table 1. Data are representative of two separate experiments.

Amphotericin B concentrations were measured in L. donovani-infected (14 or 35 days after infection) and age- and husbandry-matched uninfected BALB/c mice after administration of 2.5 mg/kg AmBisome. At both time points, amphotericin B concentrations were significantly lower (*P* ≤ 0.0001) in livers and spleens from infected BALB/c mice compared to uninfected ones. In the liver, mean drug concentrations (ng amphotericin B/g tissue) on days 21 and 42 postinfection, respectively, were 3.5- and 6.5-fold lower in infected compared to uninfected BALB/c mice. This translated into 2.5- and 5.4-fold differences when taking organ weights into account and estimating the total amount of amphotericin B per organ (Fig. 2A and B). In the spleen, mean amphotericin B concentrations were 29.3- and 100.9-fold lower, respectively, in infected compared to uninfected mice on days 21 and 42 postinfection. Respective organ weight adjusted differences were 10.3- and 16.5-fold (Fig. 2C and D). The opposite was observed in plasma, and significantly higher amphotericin B concentrations were measured in infected BALB/c mice compared to uninfected ones, with 2- and 1.9-fold differences, respectively, on day 21 (*P* ≤ 0.01) and day 42 (*P* ≤ 0.001) postinfection (Fig. 2E). Tabulated results are provided in Table S1 in the supplemental material.

To investigate if differences in amphotericin B levels were also observed after administration of higher drug doses, L. donovani-infected (day 33 postinfection) or uninfected BALB/c mice were treated with a single dose of 40 mg/kg AmBisome. In line with the above results, amphotericin B concentrations were significantly lower (*P* < 0.0001) in livers and spleens from infected BALB/c mice compared to uninfected ones. However, plasma drug concentrations were similar between the two different groups (*P* > 0.05). Data are shown in Table S1.

### Comparative amphotericin B plasma and tissue concentrations in L. donovani-infected and uninfected BALB/c mice up to 48 h after administration of single-dose AmBisome.

**FIG 3:**
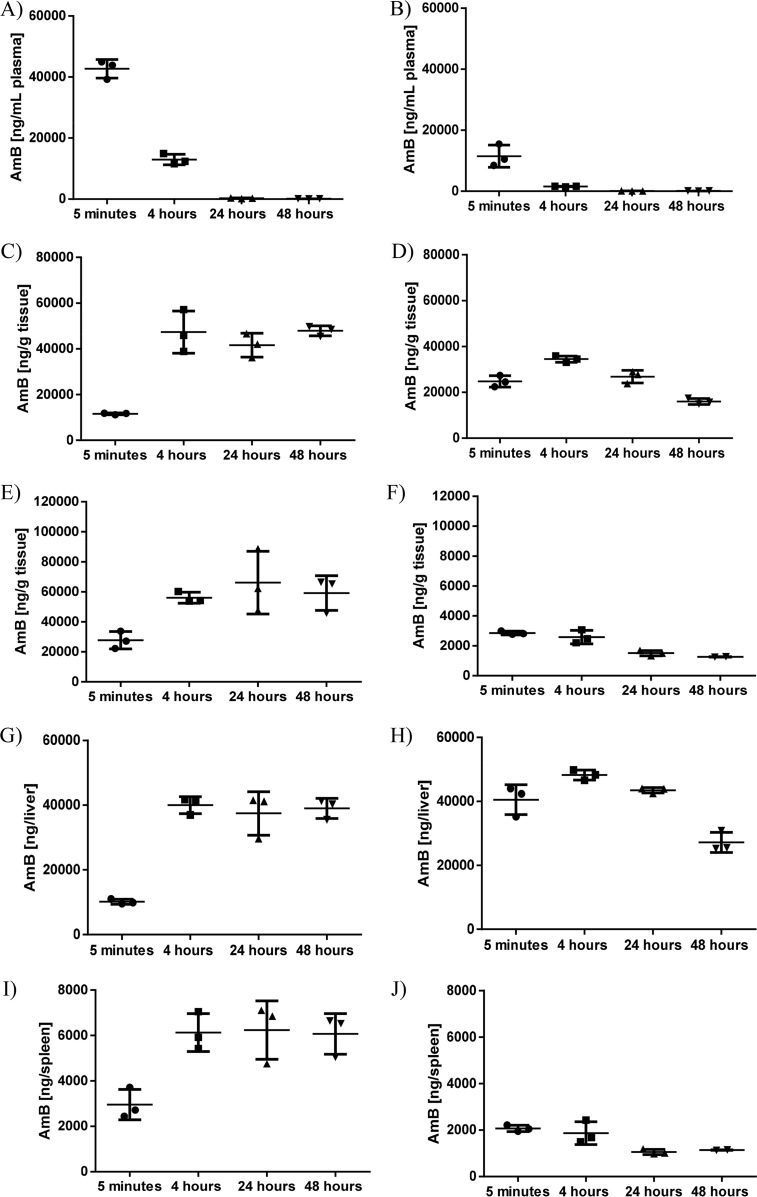
Comparative plasma and tissue drug concentrations in L. donovani-infected and uninfected BALB/c mice up to 48 h after administration of single dose AmBisome. AmBisome was administered to BALB/c mice, naive (A, C, E, G, and I) or infected with L. donovani for 33 days (B, D, F, H, and J), and amphotericin B (AmB) concentrations were determined in plasma (A and B), liver (C, D, G, and H), and spleen (E, F, I, and J) at 5 min and 4, 24, or 48 h after drug administration. Amphotericin B concentrations are presented as nanograms per gram of tissue (C to F) or total amphotericin B concentration per organ (G to J). Total amphotericin B concentrations per organ were calculated as follows: organ weight in grams (as determined at sacrifice) × amphotericin B concentration in nanograms per gram of tissue (as measured after processing of whole organs). Each symbol represents data from an individual mouse. Horizontal lines indicate the mean (*n* = 3/group) and error bars the standard deviation (SD).

A single dose of 2.5 mg/kg AmBisome was administered to L. donovani-infected mice (day 33 postinfection) and age- and husbandry-matched uninfected BALB/c mice. Plasma and tissue samples were collected 5 min and 4, 24, and 48 h after drug administration, and amphotericin B concentrations were determined. Plasma amphotericin B levels decreased within 24 h in uninfected and L. donovani-infected BALB/c mice and remained at similar levels at 24 and 48 h after drug administration (Fig. 3A and B). An increase in amphotericin B concentration was observed in livers and spleens of uninfected BALB/c mice from 5 min to 4 h after drug administration. Amphotericin B concentrations at 24 and 48 h after drug administration were comparable to those measured at 4 h (Fig. 3C and E). A different kinetic was observed in L. donovani-infected BALB/c mice. In the liver, a small increase in amphotericin B concentration was noted from 5 min to 4 h after drug administration, followed by a decrease at 24 h and, most notably, 48 h after drug administration (Fig. 3D). In the spleen, amphotericin B concentrations decreased over the whole observation period, with no increase noted (Fig. 3F). Similar kinetics were observed when estimating total amount of drug per organ (Fig. 3G to J). Tabulated results are provided in Table S2 in the supplemental material.

### Host responses to infection.

Serum samples from L. donovani-infected (days 14 and 43 postinfection) and age- and husbandry-matched uninfected BALB/c mice were subjected to electrophoresis and protein determination. Significant differences (*P* ≤ 0.05) in serum protein profiles were identified. In infected BALB/c mice, serum concentrations of total protein (46.8 ± 0.6 versus 56.6 ± 1.0 g/liter), alpha globulin (6.7 ± 0.2 versus 8.5 ± 0.2 g/liter), and gamma globulin (7.6 ± 0.2 versus 16.1 ± 1.0 g/liter; 16.3% ± 0.5% versus 28.4% ± 1.3%) increased from day 14 to day 43 postinfection. At the same time, percentages of albumin decreased (61.4% ± 0.7% versus 49.3% ± 1.1%), but no significant difference was found when comparing total albumin levels. The albumin/globulin ratio decreased from day 14 to day 43 postinfection (1.6 ± 0.0 versus 1.0 ± 0.0). Compared to uninfected BALB/c mice, higher levels of total protein (56.6 ± 1.0 versus 47.6 ± 0.8 g/liter), alpha globulin (8.5 ± 0.2 versus 7.3 ± 0.2 g/liter), and gamma globulin (16.1 ± 1.0 versus 6.5 ± 0.2 g/liter; 28.4% ± 1.3% versus 13.6% ± 0.2%) were measured in L. donovani-infected mice on day 43 postinfection. At the same time point, infected BALB/c mice had lower levels of albumin (27.8 ± 0.4 versus 29.9 ± 0.5 g/liter; 49.3% ± 1.1% versus 62.8% ± 1.6%) than uninfected ones. On day 14 postinfection, increased percentages of gamma globulin were noted in infected compared to uninfected BALB/c mice (16.3% ± 0.5% versus 12.3% ± 0.6%). On day 43 postinfection, infected BALB/c mice had a lower albumin/globulin ratio than uninfected ones (1.0 ± 0.0 versus 1.7 ± 0.1). Data are summarized in Table 5.

**TABLE 5:** Serum protein profile in L. donovani-infected and uninfected BALB/c mice^*a*^

Parameter	Result for parameter shown			
	Day 14		Day 43	
	Infected	Uninfected	Infected	Uninfected
Total protein (g/liter)	46.8 ± 0.6 A	46.2 ± 0.6	56.6 ± 1.0 AB	47.6 ± 0.8 B
Albumin				
g/liter	28.7 ± 0.4	29.5 ± 0.6	27.8 ± 0.4 B	29.9 ± 0.5 B
%	61.4 ± 0.7 A	64.0 ± 1.1	49.3 ± 1.1 AB	62.8 ± 1.6 B
Alpha globulin				
g/liter	6.7 ± 0.2 A	6.8 ± 0.2	8.5 ± 0.2 AB	7.3 ± 0.2 B
%	14.4 ± 0.3	14.7 ± 0.4	15.1 ± 0.3	15.3 ± 0.3
Beta globulin				
g/liter	3.7 ± 0.3	4.2 ± 0.4	4.1 ± 0.1	4.0 ± 0.8
%	7.9 ± 0.5	9.0 ± 0.7	7.2 ± 0.2	8.2 ± 1.6
Gamma globulin				
g/liter	7.6 ± 0.2 A	5.7 ± 0.3	16.1 ± 1.0 AB	6.5 ± 0.2 B
%	16.3 ± 0.5 AC	12.3 ± 0.6 C	28.4 ± 1.3 AB	13.6 ± 0.2 B
Albumin/globulin ratio	1.6 ± 0.0 A	1.8 ± 0.1	1.0 ± 0.0 AB	1.7 ± 0.1 B

a BALB/c mice (*n* = 6 or 7/group) were infected with L. donovani for 14 days or 43 days or left uninfected but maintained under identical conditions to infected mice. Data are presented as mean ± SEM. The parasite burdens (mean ± SD) in infected livers were 396 ± 61 and 273 ± 52 LDU on days 14 and 43 postinfection, respectively, and those in infected spleens were 8 ± 2 and 93 ± 18 LDU, respectively. Values indicated with the same letter (A, B, or C) are significantly different (*P* ≤ 0.05).

## DISCUSSION

The pharmacodynamics (PD) and pharmacokinetics (PK) of current antileishmanial drugs have yet to be fully established in preclinical disease models. Here we investigated the potency and biodistribution of liposomal amphotericin B (AmBisome) in EVL after administration of three different single doses and at two different time points. AmBisome was well tolerated at all doses administered, and no signs of drug toxicity were noted, based on observations of weight, fur condition, behavior, and facial expression. We also determined the PK profile of AmBisome in plasma of L. donovani-infected BALB/c mice in an exploratory study and compared trends to published data in uninfected mice. The observed increase in maximum concentration of drug in serum (*C*_max_) and AUC_last_ and the decrease in *V* and CL with increasing doses are in line with these data (7).

After infection of BALB/c mice with L. donovani, the parasite burden in the liver rapidly increases for the first 2 to 4 weeks, followed by a decrease in parasite numbers and resolution of infection. In the spleen, the parasite burden increases from 2 weeks after infection and parasite persistence is observed (13–15). Consideration of this organ-specific pattern of parasite growth is important when analyzing drug efficacy and potency data. In the spleen, drug efficacy was evaluated against an increasing parasite burden at both time points, and a decrease in potency was observed when drug was administered on day 35 compared to day 14 postinfection. However, the magnitude of decrease and the dose at which this occurred differed between experiments. It is notable that the decrease in drug concentrations/total drug amounts in the spleen following administration of the same dose of AmBisome on day 14 versus day 35 postinfection was less pronounced in the third compared to the second experiment presented in Table 1 (see Table S3 in the supplemental material). This may explain the lack of decreased drug potency observed in experiment 3. In the liver, opposing trends were observed at the lowest dose. However, data obtained in the liver on day 42 postinfection are not indicative of direct drug kill only: the data reflect a mixture of parasite killing by drug and a mature granulomatous host response resolving the infection (16–18). Hence, these opposing trends at a dose that lacked meaningful antileishmanial drug efficacy likely reflect differences in the efficiency of host response rather than in drug potency.

In parallel to evaluating drug potencies, we determined amphotericin B concentrations in plasma, livers, and spleens of infected BALB/c mice at the two different time points and estimated the total drug amount per organ. In livers and spleens, lower drug concentrations and total drug amounts per organ were noted when drug was administered on day 35 compared to day 14 postinfection, and the difference was highest at the lowest dose. The magnitude of difference was lower for absolute (organ weight adjusted) values than for relative values, which suggests that differences in drug levels between the two time points can partly be explained by the infection-associated increase in organ weights. A combined effect of organ enlargement and other pathology-associated factors on drug distribution following administration of AmBisome to L. donovani-infected BALB/c mice is also supported by comparative studies in infected and uninfected mice. Again the magnitude of decrease in amphotericin B levels in livers and spleens of infected mice was lower when organ weight adjustment was applied but remained significant and increased from day 21 to day 42 postinfection in both organs. Interestingly, plasma levels of amphotericin B were higher in infected compared to uninfected mice at this point.

To gain further insight into the kinetics of parasite kill and drug distribution, we next examined drug potency and distribution at multiple and earlier time points after drug administration. The highest dose of 10 mg/kg AmBisome exerted maximum kill within 48 h of drug administration. This window of time is consistent with the drug distribution from blood to tissues within 24 h of drug administration, also observed in uninfected mice and other disease models (19, 20), and a high rate of parasite kill. Differences in amphotericin B levels between L. donovani-infected and uninfected tissues were also observed at earlier time points. Importantly, these were indicative of organ-specific drug distribution and elimination in EVL. Most notable was a lack of drug accumulation over time in L. donovani-infected spleens. It is currently not known to what extent the liposomal formulation contributes to the observed differences. Comparative biodistribution studies between single-dose AmBisome and Fungizone, another clinically used nonliposomal amphotericin B formulation, are hampered by the toxicity of Fungizone. The maximum tolerated dose of 1 mg/kg (intravenous [i.v.] bolus administration) of this formulation precludes dosing at clinically meaningful dose levels (21).

Changes in drug distribution under pathological conditions have been reported for a number of antimicrobials (9, 11, 12, 20, 22–25), and increased drug concentrations at inflammatory sites are believed to result from capillary endothelial damage and recruitment of drug-containing phagocytic cells to sites of infection. However, tissue penetration of drugs is governed by a number of factors, which include drug formulation, plasma protein binding, and underlying disease (12). In EVL, different pathophysiological features are observed in the liver and spleen. In the liver, a Th1-dominated granulomatous response is characterized by an influx of T cells, B cells, NK cells, and monocytes, with a peak in the inflammatory response around 4 weeks after infection (16–18). Kupffer cells (KCs), which in uninfected mice line the sinusoids and form a uniformly distributed phagocytic network, are recruited into the core of the granuloma in infected mice. Isolated KCs remaining in the sinusoidal network of infected mice have a reduced cell volume (26), and loss of membrane activity of KCs has been reported within 2 h of infection with L. donovani (27). Infection of the spleen is characterized by a breakdown of marginal zone architecture, loss of marginal zone macrophages (MZMs) and repositioning of marginal metallophilic macrophages (MMMs) (28), an increase in the number of red pulp macrophages (29), destruction of the follicular dendritic cell and the gp38^+^ fibroblastic reticular cell networks (13), and substantial changes to the vascular network, including sprouting of α-SMA^+^ vessels with active endothelial cell proliferation (30).

Following intravenous administration, liposomes interact with blood proteins, and an inverse relationship between the amount of protein bound and liposome clearance rate from blood has been demonstrated (31). Here we show that chronically infected BALB/c mice display an increase in serum gamma globulin and, to a lesser extent, alpha globulin and a decrease in albumin. Some of these proteins have been implicated in lipid-protein interactions (32, 33), which may account for different rates in the distribution of AmBisome from blood to tissues between infected and uninfected mice. From the bloodstream, liposomes distribute to highly perfused tissues such as the liver and spleen, which regulate drug elimination (32), and cells of the mononuclear phagocyte system (MPS) play a major role in this distribution (34). In the liver also hepatocytes can participate in the uptake and metabolism of small and phosphatidylcholine-based liposomes (35, 36), which is linked to their ability to exit fenestrated vessels in this organ (32). In uninfected mice, the diameter of liver sinusoidal endothelial cell (LSEC) fenestrae is on average 99 ± 18 nm (37), which would allow AmBisome with a mean diameter of <100 nm (7) to pass through. However, fenestrae are dynamic structures, which may undergo changes in response to local external stimuli (37), and the impact of L. donovani infection on fenestration is as yet unknown. In the spleen of uninfected mice, most of the blood flows through the marginal zone (38), and MZMs display preferential uptake of clodronate liposomes over other phagocytic cells in the spleen (39). It is currently unknown if there are also differences in the extent of drug uptake between the different cell types for AmBisome. Future studies, utilizing advanced techniques to simultaneously image drug and cells, are needed to shed light on the spatial distribution of drug in healthy and diseased organs over time (40).

Gershkovich et al. (9) hypothesized that reduced phagocytic activity of macrophages in EVL or increased drug elimination, either through leaking capillaries in inflamed tissue or binding of drug to fragments of killed parasites, may explain the differences in drug levels between infected and uninfected tissues. While the latter hypothesis cannot be ruled out, it is unlikely to fully account for the lack of drug accumulation in the spleen. Our data favor a model in which (i) increases in organ mass lead to decreased total drug amounts/organ, (ii) increased elimination and/or metabolism is the predominant feature in the liver, and (iii) drug distribution to the spleen is progressively decreased during EVL.

A retrospective analysis of risk factors for VL relapse following treatment of patients in India with 20 mg/kg AmBisome, administered as 4 doses of 5 mg/kg, showed that a slower decrease in splenomegaly during treatment, but not spleen size at admission, was significantly associated with relapse (41). In L. donovani-infected BALB/c mice, the granulomatous response in the liver appears to follow many of the characteristics observed in subclinical human infection, whereas the spleen shows hallmarks of progressive human disease (42). However, it is currently unknown if the differences we observed in AmBisome distribution and elimination in this experimental model also exist at different stages in human VL. Nonetheless, understanding the mechanisms of how the pathophysiology of EVL affects drug absorption, distribution, metabolism, and elimination (ADME) will improve the development and use of antileishmanial drugs and drug delivery systems.

## MATERIALS AND METHODS

### Drugs and reagents.

AmBisome was purchased from Gilead (Cambridge, United Kingdom). The powder was reconstituted in sterile water following the manufacturer's directions, and further dilutions were prepared in 5% glucose. Amphotericin B (Vetranal analytical standard), tolbutamide, dimethyl sulfoxide (DMSO), and sodium dodecyl sulfate (SDS) were obtained from Sigma, United Kingdom, and heparin was obtained from John Bell & Croyden, United Kingdom. Methanol (high-performance liquid chromatography [HPLC] grade), 0.1% (vol/vol) formic acid in water (liquid chromatography-mass spectrometry [LC-MS] grade), and water (LC-MS grade) were purchased from Fisher Scientific UK, Ltd., United Kingdom.

### *In vivo* experiments and treatment.

Female BALB/c (Charles River, United Kingdom) and Rag-1 (B6) knockout (KO) mice (London School of Hygiene & Tropical Medicine [LSHTM] breeding colony) were maintained under specific-pathogen-free conditions in individually ventilated cages and exposed to 12-h-light–12-h-dark cycles. Standard rodent diet (RM no. 1 expanded) and filtered tap water were supplied *ad libitum*. Mice (6 to 10 weeks of age at the start of experiments) were infected by intravenous (i.v.) injection of 2 × 10^7^ parasites (L. donovani MHOM/ET/67/HU3) as described previously (43). Parasites were maintained in Rag-1 (B6) KO mice, and amastigotes were harvested from spleens >40 days after infection. BALB/c mice were treated either 14 or 35 days after infection with a single i.v. dose of AmBisome in a 0.2-ml bolus injection. On the day of treatment, prior to the administration of drugs, mice were weighed and randomized into the different treatment groups using a random number generator. The average weight of mice in each experiment was used for dose calculations. Untreated groups of mice were included as controls where appropriate. Age-matched uninfected mice were maintained under identical conditions for the same length of time as their infected comparators. At experimental endpoints, mice were weighed and humanely killed by exsanguination under terminal anesthesia. Blood was collected by cardiac puncture in Eppendorf tubes containing heparin, and plasma was harvested by centrifugation. Livers and spleens were removed, and their weight was recorded. Plasma and tissue samples were stored at −80°C until further processing. For determination of parasite burdens, tissue impression smears were prepared, fixed in 100% methanol, and stained in 10% Giemsa stain. Parasite burden was determined microscopically, and Leishman-Donovan units (LDU) were calculated by the formula no. of parasites per host cell nucleus × organ weight in mg as described previously (44). Microscopic evaluation of impression smears was carried out by a scientist unaware of the treatment allocation.

### Processing of samples for drug quantification.

Samples were thawed at room temperature immediately prior to processing. Livers and spleens were homogenized with an equal volume of zirconium oxide (ZrO) beads in 4 volumes of 0.1% aqueous formic acid in a Bullet blender (Next Advance, United Kingdom). For control matrix samples plus internal standard (IS), calibration standards, quality control (QC) samples, and study samples, 50 μl of tissue homogenate or plasma was diluted with 250 μl IS solution (200 ng/ml tolbutamide in an 84:16 [vol/vol] mixture of methanol-DMSO). For blank samples (matrix sample without IS), 50 μl of tissue homogenate or plasma was diluted with an 84:16 (vol/vol) mixture of methanol-DMSO. After shaking for 10 min at 200 rpm at room temperature, dilutions were centrifuged at 4,150 × *g* for 15 min at 4°C. Supernatants were transferred to 96-well plates and stored at −80°C.

### Preparation of calibration standards and QC samples.

An amphotericin B stock solution (1.0 mg/ml) was prepared in DMSO, and from that standard, spiking solutions were prepared by serial dilution in 1% SDS in water. Calibration standards at a minimum of 6 concentrations were prepared by mixing 5 μl of the spiking solutions with 45 μl of blank tissue homogenate or plasma, with the matrix matching that of the study samples to be analyzed. QC samples at selected concentrations were prepared in replicates in a similar fashion, and all samples were processed as described above.

### Preparation of study samples.

Concentrations of amphotericin B in study samples varied widely, depending upon dosing and sampling regimens. Those samples expected to contain high concentrations of amphotericin B were diluted with the matching control matrix by a suitable factor to reduce their concentration into a quantifiable range (i.e., within the range of calibration standard concentrations).

### LC-MS analytical conditions.

All samples were analyzed using an Agilent 1200 high-performance liquid chromatograph combined with an Agilent 6410A triple quadrupole mass spectrometer (both Agilent, United Kingdom). A mobile phase of water–0.1% formic acid (channel A) and methanol–0.1% formic acid (channel B) was used to elute sample components from a Kinetex column packed with 5-μm XB-C_18_ material (2.1 mm by 50 mm at 50°C; Phenomenex, United Kingdom). The mobile phase composition was initially 20% B, programmed to increase linearly to 90% B at 1.60 min after injection; after 0.4 min at 90% B, the composition was returned to its initial 20% B at 2.10 min postinjection. Amphotericin B was detected by monitoring the transition from *m*/*z* 906.5 to *m*/*z* 743.2, the dehydrated protonated molecule at *m*/*z* 906.5 being the most intense ion produced for this component in the ion source.

Analyte concentrations were quantified against calibration standards prepared in matched control matrix, with aliquots of sample and standard being injected typically in the range 1 to 5 μl, depending on the expected study sample concentration.

### Pharmacokinetic analysis.

Noncompartmental analysis (NCA) was performed with Phoenix Win Nonlin v6.3 (Certara, United Kingdom).

### Characterization of serum protein profiles.

Blood was collected from L. donovani-infected or uninfected BALB/c mice by cardiac puncture under terminal anesthesia and stored overnight at 4°C. Serum was harvested by centrifugation at 1,500 × *g* at 4°C for 15 min and stored at −80°C prior to analysis. Capillary electrophoresis and protein determinations were carried out by Laboklin GmbH & Co. KG (Bad Kissingen, Germany).

### Statistical analysis.

Statistical significance between two groups was evaluated by a *t* test, and that between more than two groups was analyzed by one-way analysis of variance (ANOVA) assuming a Gaussian distribution, followed by Sidak's multiple-comparison test for selected groups as applicable (GraphPad Prism 6). A *P* value of ≤0.05 was considered statistically significant.

### Ethical statement.

Experiments involving animals were carried out under license in accordance with the Animals (Scientific Procedures) Act of 1986 (UK Home Office Project Licenses PPL70/6997 and PPL70/8207) following approval by the Animal Welfare and Ethics Review Board at LSHTM.

## Supplementary Material

Supplemental material
