# Supplementary material for: Pharmacodynamics and Biodistribution of Single-Dose Liposomal Amphotericin B at Different Stages of Experimental Visceral Leishmaniasis
Source: Antimicrob Agents Chemother. 2017 Aug 24;61(9):e00497-17. doi: 10.1128/AAC.00497-17 (PMC5571318; doi:10.1128/AAC.00497-17)
Supplement: Supplemental material [file supp_61_9_e00497-17__index.html]

Supplemental material 

# Pharmacodynamics and Biodistribution of Single-Dose Liposomal Amphotericin B at Different Stages of Experimental Visceral Leishmaniasis

## Supplemental material

- Supplemental file 1 -

  Table S1, Table S2, Table S3

  PDF, 121K
