# Supplementary material for: Pharmacodynamics and Biodistribution of Single-Dose Liposomal Amphotericin B at Different Stages of Experimental Visceral Leishmaniasis
Source: Antimicrob Agents Chemother. 2017 Aug 24;61(9):e00497-17. doi: 10.1128/AAC.00497-17 (PMC5571318; doi:10.1128/AAC.00497-17)
Supplement: Supplemental material [file AAC.00497-17_zac009176474s1.pdf]

Supplementary Table S1. Comparative plasma and tissue concentrations in *L. donovani* infected and uninfected BALB/c mice 7 days after administration of single dose AmBisome®. BALB/c mice were infected with *L. donovani* and either treated with 2.5 mg/kg AmBisome® on day 14 or 35 post infection (A) or treated with 40 mg/kg AmBisome® on day 33 post infection (B). Uninfected BALB/c mice were maintained and treated under identical conditions. Seven days after drug administration groups of mice (n=5) were killed and plasma, livers and spleens harvested. Amphotericin B concentrations were determined by quality controlled LC-MS/MS techniques. Data is presented as the group mean +/- standard deviation (SD). Total amphotericin B amount / organ was calculated for individual mice as follows: organ weight in g (as determined at sacrifice) * amphotericin B concentration in ng/g tissue (as measured after processing of whole organs).

A)

	**Day 21 post infection**		**Day 42 post infection**		**Ratio Uninfected to Infected**	
	**Uninfected**	**Infected**	**Uninfected**	**Infected**	**Day 21 p.i.**	**Day 42 p.i.**
	**Amphotericin B concentration [ng/g tissue] or [ng/mL plasma], mean +/- SD**					
**Liver**	42 381 +/- 5 174	12 114 +/- 1 433	35 818 +/- 4 585	5 492 +/- 862	3.5	6.5
**Spleen**	54 038 +/- 22 545	1 844 +/- 414	62 973 +/- 17 000	624 +/- 205	29.3	100.9
**Plasma**	54 +/- 9	112 +/- 18	67 +/- 18	129 +/- 28	0.5	0.5
	**Organ weights [mg], mean +/- SD**					
**Liver**	763 +/- 17	1 068 +/- 51	830 +/- 70	1 544 +/- 86	0.7	0.5
**Spleen**	103 +/- 10	288 +/- 29	110 +/- 7	654 +/- 70	0.4	0.2

	**Estimated amphotericin B amount [ng/organ], mean +/- SD**					
**Liver**	32 368 +/- 4 516	12 907 +/- 1 308	29 516 +/- 2 228	5 492 +/- 862	2.5	5.4
**Spleen**	5 398 +/- 1 712	522 +/- 79	6 873 +/- 1 627	417 +/- 171	10.3	16.5

B)

	**Uninfected**	**Infected**	**Ratio Uninfected to Infected**	**Significance**
	**Amphotericin B concentration [ng/g tissue] or [ng/mL plasma], mean +/- SD**			
**Liver**	154 719 +/- 17 416	49 731 +/- 3 469	3.1	$p \leq 0.0001$
**Spleen**	116 706 +/- 30 609	4 059 +/- 1 285	28.8	$p \leq 0.0001$
**Plasma**	117 +/- 27	139 +/- 15	0.8	n.s.
	**Organ weights [mg], mean +/- SD**			
**Liver**	797 +/- 60	1 277 +/- 55	0.6	$p \leq 0.001$
**Spleen**	100 +/- 10	507 +/- 6	0.2	$p \leq 0.0001$
	**Estimated amphotericin B amount [ng/organ], mean +/- SD**			
**Liver**	122 648 +/- 7 869	63 598 +/- 6 983	1.9	$p \leq 0.001$
**Spleen**	11 474 +/- 1 853	2 052 +/- 623	5.6	$p \leq 0.01$

Supplementary Table S2. Comparative plasma and tissue concentrations in *L. donovani* infected and uninfected BALB/c mice up to 48 hours after administration of single dose AmBisome®. *L. donovani* infected (day 33 post infection) or age and husbandry matched uninfected BALB/c mice were treated with 2.5 mg/kg AmBisome®. Groups of mice (n=3) were killed 5 minutes, 4 hours, 24 hours or 48 hours after drug administration and amphotericin B concentrations measured in plasma, livers and spleens by quality controlled LC-MS/MS techniques. Data is presented as group mean +/- standard deviation (SD). Total amphotericin B amount / organ was calculated for individual mice as follows: organ weight in g (as determined at sacrifice) * amphotericin B concentration in ng/g tissue (as measured after processing of whole organs).

	**Minutes after drug administration:**			
	**5**	**240**	**1 440**	**2 880**
	**Amphotericin B concentration [ng/g tissue], mean +/- SD**			
**Liver uninfected**	11 577 +/- 379	47 338 +/- 9 208	41 613 +/- 5 241	47 905 +/- 2 196
**Spleen uninfected**	27 717 +/- 5 783	56 087 +/- 3 640	66 184 +/- 20 884	59 187 +/- 11 565
**Liver infected**	24 762 +/- 2 468	34 472 +/- 1 378	26 833 +/- 2 748	15 993 +/- 1 278
**Spleen infected**	2 860 +/- 122	2 586 +/- 448	1 511 +/- 178	1 261 +/- 16
	**Organ weights [mg], mean +/- SD**			
**Liver uninfected**	877 +/- 47	860 +/- 115	897 +/- 80	813 +/- 29
**Spleen uninfected**	107 +/- 6	110 +/- 20	97 +/- 15	103 +/- 6
**Liver infected**	1 637 +/- 83	1 400 +/- 10	1 633 +/- 188	1 697 +/- 81
**Spleen infected**	727 +/- 75	717 +/- 64	703 +/- 55	880 +/- 46

	**Estimated amphotericin B amount [ng/organ], mean +/- SD**			
**Liver uninfected**	10 154 +/- 761	40 013 +/- 2 637	37 452 +/- 6 737	39 004 +/- 3 106
**Spleen uninfected**	2 958 +/- 668	6 129 +/- 832	6 234 +/- 1 288	6 072 +/- 892
**Liver infected**	40 546 +/- 4 675	48 251 +/- 1 585	43 490 +/- 835	27 174 +/- 3 144
**Spleen infected**	2 072 +/- 135	1 871 +/- 494	1 060 +/- 109	1 141 +/- 12
	**Amphotericin B concentration [ng/mL plasma], mean +/- SD**			
**Plasma uninfected**	42 702 +/- 3 036	12 948 +/- 1 746	274 +/- 118	103 +/- 14
**Plasma infected**	11 498 +/- 3 602	1 555 +/- 95	99 +/- 8	114 +/- 36

Supplementary Table S3. Tissue concentrations in *L. donovani* infected BALB/c mice 7 days after administration of 2.5 mg/kg single dose AmBisome®. BALB/c mice were infected with *L. donovani* and on day 14 or 35 post infection treated with a single dose of 2.5 mg/kg AmBisome®. Seven days after drug administration groups of mice (n=4) were killed and livers and spleens harvested. Amphotericin B concentrations were determined by LC-MS/MS techniques. Data is presented as the group mean +/- standard deviation (SD). Total amphotericin B amount / organ was calculated for individual mice as follows: organ weight in g (as determined at sacrifice) * amphotericin B concentration in ng/g tissue (as measured after processing of whole organs). Experiment (Expt.) numbers refer to experiments presented in Table 1.

	**Day 21 post infection**		**Day 42 post infection**		**Ratio Day 21 vs. Day 42**	
	**Expt. 2**	**Expt. 3**	**Expt. 2**	**Expt. 3**	**Expt. 2**	**Expt. 3**
	**Amphotericin B concentration [ng/g tissue], mean +/- SD**					
**Spleen**	1 250 +/- 582	1 844 +/- 414	166 +/- 20	624 +/- 205	7.5	3.0
	**Organ weights [mg], mean +/- SD**					
**Spleen**	294 +/- 25	288 +/- 29	530 +/- 55	654 +/- 70	0.6	0.4
	**Estimated amphotericin B amount [ng/organ], mean +/- SD**					
**Spleen**	358 +/- 141	522 +/- 79	88 +/- 16	417 +/- 171	4.1	1.3
